# The Impact of Parental Monitoring on Exposure to Multiple Substances and Bullying in Croatian Students

**DOI:** 10.3390/children11111292

**Published:** 2024-10-25

**Authors:** Maja Valentic, Luka Simetin, Dijana Mayer, Filip Simetin, Ivana Pavic

**Affiliations:** 1 Croatian Institute of Public Health, 10000 Zagreb, Croatia; dijana.mayer@hzjz.hr (D.M.); ivana.pavic@hzjz.hr (I.P.); 2 School of Medicine, University of Zagreb, 10000 Zagreb, Croatia; lsimetin@mef.hr (L.S.); fsimetin@mef.hr (F.S.); 3 School of Medicine, Catholic University of Zagreb, 10000 Zagreb, Croatia

**Keywords:** parental monitoring, substance use, bullying, students

## Abstract

**Background/Objectives:** Adolescence is a critical period for experimenting with a wide range of risky behaviors, which are often influenced by family dynamics, including parental monitoring. This study aims to analyze the patterns of exposure to multiple substances and bullying among Croatian students by age and gender in 2022 and examine the association of exposure to multiple substances and bullying with maternal and paternal monitoring. **Methods**: The data were obtained from the 2022 Health Behaviour in School-aged Children (HBSC) study conducted in Croatia among students aged 11, 13, and 15. Two multinomial logistic regressions were performed separately by age and gender. **Results**: Exposure to multiple instances of bullying is more common among boys than girls in all three age groups, while exposure to multiple substances varies depending on age. The analysis revealed the strong protective effect of maternal monitoring against both substance use and bullying. Paternal monitoring showed less consistent effects but still indicated that lower paternal monitoring was associated with higher exposure to multiple substances and bullying, particularly at age 13. **Conclusions**: The patterns of multiple substance use and bullying vary by gender and age, emphasizing the need for tailored intervention strategies. Programs that strengthen parental monitoring, particularly maternal monitoring, should be prioritized.

## 1. Introduction

Adolescence is a dynamic and critical development period characterized by many social, physical, physiological, and psychological changes [1]. Its defining characteristics include independence from parents and family, valuing friendships and peer groups, exploring one’s identity, and experimenting with a wide range of behaviors, some of which are risky and inappropriate [1]. 

Adolescent multiple substance use is a significant and growing problem today, as it is linked to poor mental health as well as negative educational and social outcomes [2,3,4]. The early initiation of substance use and the use of multiple substances are strong indicators of future substance use problems and disorders [4]. Patterns of using multiple substances may identify different groups of adolescents with unique risk factors and future outlooks [2].

School bullying is a major social problem affecting children and adolescents worldwide [5]. It involves repeated negative actions over a period of time and can be direct, such as physical and verbal violence, or indirect, such as social exclusion [6]. Bullying is characterized by harmful intent and an imbalance of power that makes it hard for the victim to defend themselves [6]. A relatively new form of bullying is called cyberbullying, i.e., bullying via mobile phone or the Internet [7]. Many children who are involved in bullying, either as perpetrators or as victims, run the risk of facing psychological difficulties later in life [6,7,8,9,10,11,12,13].

Family dynamics is one of the key factors for shaping behaviors such as adolescent substance use and bullying [14,15,16]. Throughout adolescence, parents play a critical role in promoting healthy development, partly through parental monitoring [17,18,19,20]. Parental monitoring is a set of parenting behaviors that involves attention to and tracking youth’s whereabouts, activities, and friendships [21].

The existing research on adolescent substance use emphasizes the importance of parental monitoring as a protective factor in its prevention [20,22,23,24,25,26]. Some studies suggest that parental monitoring has a protective effect against bullying and its negative outcomes, while other studies indicate it may be unrelated or even positively related to bullying perpetration and victimization [18,27,28,29,30,31]. Moreover, some studies suggest that maternal monitoring or maternal knowledge reduces adolescent substance use and bullying behaviors, while other studies highlight the additional protective effects of paternal knowledge and father–youth connectedness [32,33,34,35,36].

However, research that focuses on multiple substance use as well as exposure to multiple instances of bullying and its association with parental monitoring is still missing. Besides, there is a lack of research on these dynamics in Croatia, where cultural nuances may affect these associations despite their recognized importance.

This study aims to analyze the patterns of exposure to multiple substances and multiple instances of bullying among Croatian students by age and gender in 2022. Additionally, we aim to investigate the association of exposure to multiple substances as well as multiple instances of bullying with parental monitoring, both maternal and paternal. Exposure to multiple substances and bullying reveals particularly vulnerable students, which is why it is especially important to understand the characteristics of their parents to form appropriate preventive activities.

## 2. Materials and Methods

### 2.1. Sample

The data used for analysis were obtained from the 2022 Health Behaviour in School-aged Children (HBSC) study conducted in Croatia. The HBSC study is a WHO cross-sectional study that takes place every four years in various countries across Europe and North America. It comprises data from students aged 11, 13, and 15, and follows an internationally standardized protocol.

The Croatian sample was selected based on the official list of schools provided by the Ministry of Science and Education. The sampling unit used was the school class. School classes were chosen randomly at the national level and, for 15-year-olds, they were categorized by the type of high school. The sample consisted of 5338 students, with 51.69% girls (2759) and 48.31% boys (2579), and had a response rate of 64.60%. The sample included 1763 students aged 11 (average age 11.07), 1940 aged 13 (average age 12.96), and 1635 aged 15 (average age 14.99).

### 2.2. Measures

An internationally standardized questionnaire, translated into Croatian by back-translation, was used as the research instrument. Data collection occurred in the spring of 2022, between March and May. The survey was carried out anonymously and voluntarily, with passive parental consent. The online questionnaire was self-administered by students in the classroom under the supervision of a teacher, using the LimeSurvey online platform (https://community.limesurvey.org/; Version 3.28.26+220829).

#### 2.2.1. Exposure to Multiple Substances

We created a new exposure to multiple substances variable, which we used as the dependent variable. We composed exposure to multiple substances from the following four variables at the age of 11 and 13: got drunk, smoked cigarettes, tried e-cigarettes, and drank energy drinks at least once in a lifetime. The question about lifetime cannabis use was posed only to pupils at age 15. This newly created exposure to multiple substance variable was split into three categories where those who used no substance (no exposure) were compared with those who used 1 or 2 substances (low exposure) and those who used 3 or 4 substances at the age of 11 and 13 and 3–5 substances at the age of 15 (high exposure). 

Lifetime drunkenness was assessed with the question “Have you ever drunk so much alcohol that you were really drunk in life?” Response options were on a five-point scale ranging from “never” to “more than 10 times (or more)”. 

Lifetime cigarette smoking was measured with the question “How many days did you smoke cigarettes in life?”. Response options were on a seven-point scale ranging from “never” to “30 (or more) days”. 

Lifetime e-cigarette use was evaluated using the question “How many days have you used electronic cigarettes (e.g., e-cigarettes, Wiip, e-hookah)? Please do not include products that ‘heat, not burn’ (e.g., IQOS, Glo, TEEPS)”. Response options were on a seven-point scale ranging from “never” to “30 (or more) days”.

Lifetime energy drinks use was evaluated using the question “Currently, how often do you drink energy drinks (e.g., Red Bull, Burn, Monster)? Also count taking small amounts”. Response options were on a five-point scale ranging from “never” to “every day”.

Lifetime cannabis use was evaluated using the question “Have you ever used cannabis?”. Response options were on a seven-point scale ranging from “never” to “30 (or more) days”.

#### 2.2.2. Exposure to Multiple Instances of Bullying

We formed a new exposure to multiple instances of bullying variable, which we used as the dependent variable. We determined exposure to multiple instances of bullying from the following four variables: bullying perpetration, bullying victimization, cyberbullying perpetration, and cyberbullying victimization. This newly created exposure to multiple instances of bullying variable was split into three categories, where the category “no exposure” includes those who did not participate in bullying, including cyberbullying, neither as victims nor as perpetrators. The category of “low exposure” includes the response “once or twice” regarding 1 to 4 bullying variables combined with none, and 1 response of “2 or 3 times a month”, combined with 3 negative answers. All other responses to at least one of four bullying variables (“2 or 3 times a month” in more than one bullying variable, “about once a week”, and “several times a week”) are classified in the “high exposure” category.

Bullying perpetration was assessed with the question “How often have you taken part in bullying another student(s) at school in the past couple of months?”.

Bullying victimization was measured with the question “How often have you been bullied at school in the past couple of months?”.

Cyberbullying perpetration was evaluated using the question “In the past couple of months how often have you taken part in cyberbullying (e.g., sent mean instant messages, email or text messages, wall postings, created a website making fun of someone, posted unflattering or inappropriate pictures without permission and posted them online or sent them to others)?”.

Cyberbullying victimization was assessed with the question “In the past couple of months how often have you been cyberbullied (i.e., someone sent mean instant messages, email or text messages, wall postings, created a website making fun of me or someone took unflattering or inappropriate pictures of me without permission and posted them online)?”.

Response options for all four bullying variables were not once, once or twice, two or three times a month, about once a week, and several times a week.

#### 2.2.3. Parental Monitoring

We assessed parental monitoring with two created variables: maternal and paternal monitoring. The parental monitoring variables were independent in the analysis. We derived these two new variables from the answer to an identical question about mother and father with the following sub-questions: “How much does your mother/father know about...? who your friends are, how you spend your money, where you are after school, where do you go at night, and what you do on the internet”. Response options were as follows: mother/father “knows a lot”, “knows a little”, “doesn’t know anything”, and “doesn’t have or doesn’t see mother/father”. These newly created parental monitoring variables were split into three categories. The category “high monitoring” included the responses “knows a lot” to all five sub-questions and combinations of one response “knows a little” with four responses “knows a lot”. Other responses fell into categories “low mother/father monitoring” or “no monitoring” (“don’t have or don’t see mother/father”).

### 2.3. Statistical Analysis

Descriptive statistics was used to present the sample characteristics. Gender differences in dependent and independent variables were examined and tested using Pearson’s chi-squared test. Two multinomial logistic regressions were performed separately for boys and girls in three age categories: 11, 13, and 15 years. First, a multinomial logistic regression was performed with exposure to multiple substances as a dependent variable and two mutually independent variables/factors: monitored by the mother and monitored by the father. Second, a multinomial logistic regression was performed with exposure to multiple instances of bullying as the dependent variable and two mutually independent variables/factors: monitored by the mother and monitored by the father.

The results of the logistic regression were presented as odds ratios with 95% confidence intervals.

The statistical significance level was set at *p* < 0.05.

IBM SPSS version 28 (IBM, Armonk, NY, USA) was used for conducting the statistical analyses.

## 3. Results

The results section is organized as follows: first, we present the findings related to exposure to multiple substances by age and gender, followed by the analysis of exposure to multiple instances of bullying. Lastly, we examine the associations between parental monitoring and exposure to both substances and bullying.

As presented in Table 1, high exposure to multiple substances was more prevalent among boys at age 11 (6.6%) compared to girls (4.6%), with a significant gender difference (*p* < 0.001). By age 15, girls started to show higher exposure than boys (40.5% vs. 34.2%, *p* = 0.025). Substance use increased with age for both genders, with girls surpassing boys in cigarette (42.7% vs. 34.8%) and e-cigarette (42.7% vs. 34.8%) use at age 15 (*p* < 0.001 for both). Drunkenness was more common among boys at age 11 (11.9% vs. 7.1%, *p* = 0.001), but by age 15, the rates were almost identical for both genders (45.3% vs. 45.9%). Similarly, boys at age 11 were more likely to consume energy drinks (33.4% vs. 25.6%, *p* < 0.001), but by age 15, the gender gap narrowed (64.4% of boys and 61.1% of girls). For cannabis use, no significant gender differences were found at age 15 (15.9% for boys and 16.5% for girls).

As presented in Table 2, exposure to bullying was more frequent among boys at age 11 (9.4% vs. 6.4%, *p* < 0.001), and boys remained more involved in bullying perpetration and victimization across all age groups. At age 15, boys were more likely to be victims of bullying 2–3 times per month (10.8% vs. 5.3%, *p* < 0.002), and cyberbullying perpetration was higher among boys compared to girls at age 15 as well (7.3% vs. 1.8%, *p* < 0.001).

As shown in Table 3, at age 11, 34.0% of boys reported high maternal monitoring compared to 27.1% of girls (*p* = 0.004). By age 15, the figures increased to 53.5% for boys and 44.9% for girls (*p* < 0.001). More girls than boys at age 15 reported that their mothers knew about their friends (76.5% vs. 66.9%, *p* < 0.001). Moreover, 85.3% of boys and 90.8% of girls at age 11 stated their mothers knew where they went at night (*p* < 0.001), decreasing to 71.6% for boys and 81% for girls at age 15 (*p* < 0.001). When it comes to spending money, 62.8% of boys and 71.2% of girls aged 15 stated that their mother knows a lot about how they spend their money (*p* = 0.005). More girls compared to boys reported that the mother knows about where they are after school at the age of 11 (89.5% vs. 84.7%, *p* = 0.002) and at the age of 15 (79.7% vs. 73.6%, *p* = 0.022). In terms of internet use, statistical significance was found at the age of 11 (53.8% of boys and 58.6% of girls) and the age of 13 (33.6% of boys and 37.9% of girls, *p* = 0.019). For paternal monitoring, high monitoring was indicated by 50.5% of boys and 50.1% of girls at age 11, with a statistically significant difference found at age 13, where 54.0% of boys reported high monitoring compared to 60.4% of girls (*p* = 0.024).

More boys than girls at age 11 reported that their fathers knew about their friends (67.3% vs. 60.1%, *p* = 0.001), with 54.5% of boys and 45.3% of girls at age 15 indicating the same (*p* < 0.001). At age 15, 54.4% of boys and 47.6% of girls responded that their fathers know how they spend their money (*p* = 0.005). Also, more 13-year-old boys compared to girls stated that their father knows about where they are after school (62.5% vs. 57.9%, *p* = 0.036). Regarding paternal monitoring and internet use, statistical significance was found at the age of 13 (32.5% of boys and 28.6% of girls, *p* = 0.030) and age 15 (26.2% of boys and 22.1% of girls, *p* = 0.026).

Table 4 shows that boys aged 11 with no maternal monitoring had 23.21 times higher odds (CI 4.71–114.32) of high substance exposure compared to those with high maternal monitoring. Low maternal monitoring was also associated with increased odds for both high (4.23, CI 1.63–10.95) and low substance exposure (2.19, CI 1.46–3.27).

Among 13-year-old boys, low maternal monitoring was associated with 3.23 times higher odds of high substance exposure (CI 1.81–5.78), while no monitoring was linked to 3.11 times higher odds of low substance exposure (CI 1.09–8.83). Low maternal monitoring also increased the odds of low substance exposure (OR 1.82, CI 1.25–2.64). 

For 15-year-old boys, no maternal monitoring resulted in 4.63 times higher odds of high substance exposure (CI 1.34–16.01), and low monitoring led to 2.74 times higher odds (CI 1.48–5.06).

In 11-year-old girls, low maternal monitoring increased the odds of high substance exposure by 4.65 times (CI 1.30–16.58) and, for low exposure, by 1.94 times (CI 1.30–2.91). No paternal monitoring was linked to 2.64 times higher odds for low substance exposure (CI 1.07–6.56), and low paternal monitoring was associated with a 1.65 times increase (CI 1.11–2.44).

For 13-year-old girls, high odds of substance exposure were linked to no maternal monitoring (OR 14.04, CI 2.61–75.55), low maternal (OR 3.87, CI 2.40–6.25), no paternal (OR 2.81, CI 1.10–7.25), and low paternal monitoring (OR 2.27, CI 1.27–4.06). Higher odds of low exposure were associated with low maternal (OR 2.08, CI 1.46–2.96) and low paternal monitoring (OR 1.43, CI 1.01–2.02).

For girls aged 15, higher odds of high substance exposure were linked to low maternal monitoring (OR 3.90, CI 2.48–6.13), no paternal monitoring (OR 5.08, CI 1.94–13.32), and low paternal monitoring (OR 1.87, CI 1.16–2.99). Low maternal monitoring was also associated with higher odds of low substance exposure (OR 2.16, CI 1.37–3.40). 

As shown in Table 5, in 11-year-old boys, low maternal monitoring was associated with a 4.28 times increase (CI 2.11–8.67) in exposure to bullying, while no paternal monitoring increased odds by 6.85 times (CI 2.15–21.81). Low maternal monitoring was linked to a 2.35 times higher risk (CI 1.54–3.58) for low exposure to bullying.

Among boys aged 13, no maternal monitoring was associated with 10.32 higher odds (CI 2.66–40.13), and low maternal monitoring with 2.34 higher odds (CI 1.21–4.50) for high exposure to bullying. Similarly, no paternal monitoring was linked to 3.29 higher odds (CI 1.06–10.25), and low paternal monitoring to 1.48 higher odds (CI 1.01–2.18) for low exposure to bullying.

For boys aged 15, no maternal monitoring was associated with 9.06 higher odds (CI 2.78–29.47), and low maternal monitoring with 3.09 higher odds (CI 1.33–7.18) for high exposure to bullying.

Among girls aged 11, low maternal monitoring was associated with 2.09 higher odds (CI 1.01–4.36), no paternal monitoring with 4.54 higher odds (CI 1.05–19.68), and low paternal monitoring with 2.54 higher odds (CI 1.15–5.61) for high exposure to bullying. Additionally, low maternal monitoring was associated with 1.83 higher odds (CI 1.21–2.78), and low paternal monitoring with 1.66 higher odds (CI 1.14–2.44) for low exposure to bullying.

For girls aged 13, no maternal monitoring was associated with 16.13 higher odds (CI 3.29–79.01), and low maternal monitoring with 3.13 higher odds (CI 1.65-5.29) for high exposure to bullying. No paternal monitoring increased the odds by 5.80 (CI 1.54–21.88), while low paternal monitoring increased the odds by 3.14 (CI 1.32–7.45). For low exposure to bullying, low maternal monitoring was associated with 1.91 higher odds (CI 1.35–2.71), no paternal monitoring with 4.00 higher odds (CI 1.85–8.64), and low paternal monitoring with 1.89 higher odds (CI 1.31–2.73).

Among girls aged 15, low exposure to bullying was associated with 5.19 higher odds (CI 1.70–15.84) for no maternal monitoring, 1.92 higher odds (CI 1.33–1.77) for low maternal monitoring, 2.94 higher odds (CI 1.52–5.68) for no paternal monitoring, and 2.11 higher odds (CI 1.34–3.33) for low paternal monitoring.

## 4. Discussion

This study examined distinct age patterns in exposure to multiple substances and bullying among boys and girls in Croatia. The proportions of multiple substance use increased with age for both boys and girls, while exposure to multiple instances of bullying is most common at the age of 13. Exposure to multiple instances of bullying is more common among boys than girls in all three age groups, while there is no clear gender pattern with exposure to multiple substances, which varies depending on age.

Even at the age of 11, a significant proportion of girls, and especially boys, are already exposed to multiple psychoactive substances and bullying. This indicates that prevention programs are already necessary in the lower grades of elementary school and that these programs should be comprehensive, rather than focused on a single substance or one form of violence.

The results of this study reveal significant associations between parental monitoring (both maternal and paternal) and multiple substance use as well as exposure to bullying among adolescents in Croatia. Patterns of substance use and bullying vary by gender and age, emphasizing the need for gender- and age-specific intervention strategies.

This study showed the strong protective effect of parental monitoring against both substance use and bullying, which aligns with previous research [18,20,22,23,24,25,26,28,29,31]. High levels of maternal monitoring were consistently associated with lower odds of both exposure to multiple substances and bullying involvement, confirming the critical role mothers play in adolescent behavioral development [37,38]. The findings are consistent with previous research showing that when mothers are more involved and emotionally connected with their children, they have a greater influence on reducing risky behaviors [32,33,34,36].

On the other hand, the level of paternal monitoring appeared to be more inconsistent. Although high paternal monitoring was generally associated with reduced exposure to substances and bullying, the protective effect was less consistent compared to maternal monitoring. This might be due to the traditional roles and expectations of mothers and fathers in Croatian society, where mothers may take on a more active role in adolescent everyday activities [39,40]. However, the strong association between the absence of paternal monitoring and higher substance use and bullying at the age of 13 indicates that fathers’ involvement becomes particularly crucial during early adolescence when adolescents are more susceptible to peer influence and external pressures [41].

## 5. Conclusions

This study shows the need for designing preventive strategies to reduce substance use and bullying among adolescents. Programs that strengthen parental monitoring, particularly maternal monitoring, should be prioritized. However, the results also indicate a need for greater paternal involvement, especially as children enter adolescence. Parenting interventions should encourage fathers to actively monitor their children’s social interactions and online activities to reduce risky behaviors.

It is demonstrated that boys and girls have different needs when it comes to preventing substance use and bullying, so interventions need to be designed specifically for each gender. For example, girls might need programs that address cigarette and e-cigarette use during their late adolescence, while boys might benefit more from earlier interventions aimed at reducing alcohol and energy drink consumption.

While this study provides valuable insights by focusing on the combined effects of multiple risk behaviors (substance use and bullying) and their associations with parental monitoring, it is not without limitations. This study relies on self-reported data, which may be subject to reporting bias and memory inaccuracies. The cross-sectional design of the research disables establishing causal relationships between variables. Future research could use longitudinal designs to track changes in parental monitoring and adolescent behavior over time.

## Figures and Tables

**Table 1:** Exposure to multiple substances by age and gender.

Exposure to Multiple Substances	Age 11				Age 13				Age 15			
	Boys	Girls	χ^2^	*p*	Boys	Girls	χ^2^	*p*	Boys	Girls	χ^2^	*p*
Exposure to multiple substances												
High exposure	58	41	16	<0.001	160	180	1.1	0.580	256	359	7.5	0.025
	6.6%	4.6%			16.7%	18.3%			34.2%	40.5%		
Low exposure	279	223			392	385			317	326		
	32.0%	25.1%			40.9%	39.2%			42.4%	36.8%		
No exposure	536	626			406	416			175	202		
	61.4%	70.3%			42.4%	42.4%			23.4%	22.8%		
Lifetime drunkenness												
At least once	104	63	12	0.001	202	185	1.5	0.220	339	407	0.1	0.820
	11.9%	7.1%			21.1%	18.9%			45.3%	45.9%		
Never	769	827			8	796			409	480		
	88.1%	92.9%			78.9%	81.1%			54.7%	54.1%		
Lifetime cigarette smoking												
At least once	72	55	2.8	0.093	171	197	1.6	0.21	260	379	10.8	0.001
	8.2%	6.2%			17.8%	20.1%			34.8%	42.7%		
Never	801	835			787	784			488	508		
	91.8%	93.8%			82.2%	79.9%			65.2%	57.3%		
Lifetime e-cigarette use												
At least once	75	63	1.4	0.237	204	239	2.6	0.108	265	410	19.5	<0.001
	8.6%	7.1%			21.3%	24.4%			35.4%	46.2%		
Never	798	827			754	742			483	477		
	91.4%	92.9%			78.7%	75.6%			64.6%	53.8%		
Lifetime energy drink use												
At least once	292	228	12.9	<0.001	490	497	0.1	0.831	482	542	1.9	0.165
	33.4%	25.6%			51.1%	50.7%			64.4%	61.1%		
Never	581	662			468	484			266	345		
	66.6%	74.4%			48.9%	49.3%			35.6%	38.9%		
Lifetime cannabis use												
At least once									119	146	0.1	0.763
									15.9%	16.5%		
Never									629	741		
									84.1%	83.5%		

**Table 2:** Exposure to multiple instances of bullying by age and gender.

Exposure to Multiple Instances of Bullying	Age 11				Age 13				Age 15			
	Boys	Girls	χ^2^	*p*	Boys	Girls	χ^2^	*p*	Boys	Girls	χ^2^	*p*
Exposure to multiple instances of bullying												
High exposure	69	49	23.5	<0.001	95	72	23	<0.001	73	31	40	<0.001
	9.4%	6.4%			11.7%	8.2%			11.3%	3.8%		
Low exposure	307	249			377	338			216	230		
	41.9%	32.5%			46.5%	38.7%			33.4%	28.4%		
No exposure	357	467			338	464			357	549		
	48.7%	61.0%			41.7%	53.1%			55.3%	67.8%		
Bullying perpetration												
Never	649	713	13.7	0.008	675	778	19	0.001	558	761	42	<0.001
	81.5%	87.3%			77.5%	85.4%			81.7%	91.8%		
Once or twice per month	107	68			121	84			58	43		
	13.4%	8.3%			13.9%	9.2%			8.5%	5.2%		
2–3 times per month or more	40	36			75	49			67	25		
	5.0%	4.4%			8.6%	5.4%			9.8%	3.0%		
Bullying victimization												
Never	613	634	1.6	0.816	656	656	5.7	0.219	555	707	17	0.002
	77.1%	77.8%			75.7%	72.2%			81.3%	84.9%		
Once or twice per month	99	101			107	143			54	82		
	12.5%	12.4%			12.3%	15.7%			7.9%	9.8%		
2–3 times per month or more	83	80			104	109			74	44		
	10.4%	9.8%			12.0%	12.0%			10.8%	5.3%		
Cyberbullying perpetration												
Never	659	731	12	0.018	650	781	27	<0.001	537	754	42	<0.001
	87.3%	91.9%			79.5%	87.6%			81.7%	92.0%		
Once or twice per month	67	42			117	93			72	51		
	8.9%	5.3%			14.3%	10.4%			11.0%	6.2%		
2–3 times per month or more	29	22			51	18			48	15		
	3.8%	2.8%			6.2%	2.0%			7.3%	1.8%		
Cyberbullying victimization												
Never	682	709	0.3	0.99	703	727	13	0.014	562	706	16	0.003
	86.5%	86.4%			81.5%	79.9%			81.8%	85.0%		
Once or twice per month	59	62			82	118			51	80		
	7.5%	7.6%			9.5%	13.0%			7.4%	9.6%		
2–3 times per month or more	47	50			78	65			74	45		
	6.0%	6.1%			9.0%	7.1%			10.8%	5.4%		

**Table 3:** Parental monitoring by age and gender.

Parental Monitoring	Age 11				Age 13				Age 15			
	Boys	Girls	χ^2^	*p*	Boys	Girls	χ^2^	*p*	Boys	Girls	χ^2^	*p*
Maternal monitoring												
High monitoring	249	214	11.3	0.004	378	342	16	<0.001	356	369	24	<0.001
	34.0%	27.1%			45.4%	38.4%			53.5%	44.9%		
Low monitoring	465	565			429	536			279	436		
	63.5%	71.4%			51.6%	60.2%			41.9%	53.1%		
No monitoring	18	12			25	13			31	16		
	2.5%	1.5%			3.0%	1.5%			4.7%	1.9%		
Mother knows a lot about…												
Who student’s friends are	632	708	6.1	0.109	646	744	11	0.011	448	629	22	<0.001
	84.3%	88.5%			76.3%	82.6%			66.9%	76.5%		
How student spends his/her money	541	613	6.9	0.074	581	647	5.4	0.148	422	586	13	0.005
	72.6%	76.7%			69.0%	72.1%			62.8%	71.2%		
Where student is after school	629	714	14.9	0.002	676	746	4.9	0.178	493	656	9.6	0.022
	84.7%	89.5%			80.3%	83.2%			73.6%	79.7%		
Where student goes at night	631	724	18.7	<0.001	675	753	6.4	0.094	480	666	19	<0.001
	85.3%	90.8%			80.1%	83.9%			71.6%	81.0%		
What student does on the internet	401	468	9.4	0.024	283	341	9.9	0.019	186	249	6.9	0.077
	53.8%	58.6%			33.6%	37.9%			27.6%	30.3%		
Paternal monitoring												
High monitoring	369	393	3.9	0.146	450	537	7.4	0.024	381	503	3.1	0.213
	50.5%	50.1%			54.0%	60.4%			57.6%	61.3%		
Low monitoring	327	369			340	308			235	256		
	44.7%	47.0%			40.8%	34.6%			35.5%	31.2%		
No monitoring	35	23			43	44			46	62		
	4.8%	2.9%			5.2%	4.9%			6.9%	7.6%		
Father knows a lot about…												
Who student’s friends are	505	483	17.4	0.001	496	430	22	<0.001	364	373	19	<0.001
	67.3%	60.1%			58.2%	47.7%			54.5%	45.3%		
How student spends his/her money	459	481	0,8	0.838	481	471	11	0.010	364	393	13	0.005
	61.5%	60.4%			56.9%	52.4%			54.4%	47.6%		
Where student is after school	510	552	4.2	0.238	529	518	8.5	0.036	380	424	7.6	0.054
	68.5%	69.1%			62.5%	57.9%			56.9%	51.5%		
Where student goes at night	534	587	5.2	0.158	560	567	7.9	0.048	390	480	3.9	0.270
	72.1%	74.1%			66.5%	63.3%			58.3%	58.2%		
What student does on the internet	355	382	2.4	0.493	275	256	9	0.030	176	182	9.2	0.026
	47.7%	48.0%			32.5%	28.6%			26.2%	22.1%		

**Table 4:** Multinomial logistic regressions by age and gender on the association between parental monitoring and exposure to multiple substances.

Age	Substance Exposure	Parental Monitoring	Boys				Girls			
			Sig.	OR	95% Confidence Interval		Sig.	OR	95% Confidence Interval	
					Lower Bound	Upper Bound			Lower Bound	Upper Bound
11	High vs. no substance exposure	No vs. high maternal monitoring	0	23.21	4.71	114.32	0.07	1.23	0.98	1.53
		Low vs. high maternal monitoring	0	4.23	1.63	10.95	0.02	4.65	1.3	16.58
		No vs. high paternal monitoring	0.99	1.01	0.2	5.05	0.12	1.08	0.98	1.2
		Low vs. high paternal monitoring	0.49	0.72	0.28	1.84	0.25	2.4	0.54	10.65
	Low vs. no substance exposure	No vs. high maternal monitoring	0.36	1.82	0.5	6.61	0.76	0.78	0.15	4.01
		Low vs. high maternal monitoring	0	2.19	1.46	3.27	0	1.94	1.3	2.91
		No vs. high paternal monitoring	0.36	1.48	0.64	3.43	0.04	2.64	1.07	6.56
		Low vs. high paternal monitoring	0.88	1.03	0.69	1.54	0.01	1.65	1.11	2.44
13	High vs. no substance exposure	No vs. high maternal monitoring	0.08	3.52	0.85	14.55	0	14.04	2.61	75.55
		Low vs. high maternal monitoring	0	3.23	1.81	5.78	0	3.87	2.4	6.25
		No vs. high paternal monitoring	0.2	1.98	0.7	5.61	0.03	2.81	1.1	7.25
		Low vs. high paternal monitoring	0.31	1.38	0.74	2.56	0.01	2.27	1.27	4.06
	Low vs. no substance exposure	No vs. high maternal monitoring	0.03	3.11	1.09	8.83	0.11	3.9	0.73	20.82
		Low vs. high maternal monitoring	0	1.82	1.25	2.64	0	2.08	1.46	2.96
		No vs. high paternal monitoring	0.78	0.89	0.41	1.95	0.96	0.98	0.45	2.13
		Low vs. high paternal monitoring	0.96	0.99	0.68	1.45	0.05	1.43	1.01	2.02
15	High vs. no substance exposure	No vs. high maternal monitoring	0.02	4.63	1.34	16.01	0.27	2.25	0.54	9.44
		Low vs. high maternal monitoring	0	2.74	1.48	5.06	0	3.9	2.48	6.13
		No vs. high paternal monitoring	0.12	2.41	0.79	7.39	0	5.08	1.94	13.32
		Low vs. high paternal monitoring	0.31	0.72	0.39	1.35	0.01	1.87	1.16	2.99
	Low vs. no substance exposure	No vs. high maternal monitoring	0.73	0.79	0.21	3.01	0.43	1.74	0.44	6.91
		Low vs. high maternal monitoring	0.7	1.11	0.65	1.91	0	2.16	1.37	3.4
		No vs. high paternal monitoring	0.13	2.36	0.78	7.19	0.06	2.48	0.96	6.39
		Low vs. high paternal monitoring	0.22	1.41	0.81	2.45	0.73	0.93	0.61	1.42

**Table 5:** Multinomial logistic regressions by age and gender on the association between parental monitoring and exposure to multiple instances of bullying.

Age	Exposure to Bullying	Parental Monitoring	Boys				Girls			
			Sig.	OR	95% Confidence Interval		Sig.	OR	95% Confidence Interval	
					Lower Bound	Upper Bound			Lower Bound	Upper Bound
11	High vs. no exposure to bullying	No vs. high maternal monitoring	0.1	3.23	0.76	13.71	0.5	2.27	0.21	23.99
		Low vs. high maternal monitoring	0	4.28	2.11	8.67	0.05	2.09	1.01	4.36
		No vs. high paternal monitoring	0	6.85	2.15	21.81	0.04	4.54	1.05	19.68
		Low vs. high paternal monitoring	0.9	1.02	0.5	2.1	0.02	2.54	1.15	5.61
	Low vs. no exposure to bullying	No vs. high maternal monitoring	0.3	0.39	0.07	2.08	0.61	1.51	0.32	7.14
		Low vs. high maternal monitoring	0	2.35	1.54	3.58	0	1.83	1.21	2.78
		No vs. high paternal monitoring	0.3	1.82	0.64	5.16	0.15	2	0.78	5.13
		Low vs. high paternal monitoring	0.3	1.24	0.83	1.84	0.01	1.66	1.14	2.44
13	High vs. no exposure to bullying	No vs. high maternal monitoring	0	10.32	2.66	40.13	0	16.13	3.29	79.01
		Low vs. high maternal monitoring	0	2.34	1.21	4.5	0	3.13	1.65	5.92
		No vs. high paternal monitoring	0	3.29	1.06	10.25	0.01	5.8	1.54	21.88
		Low vs. high paternal monitoring	0.1	1.68	0.84	3.36	0.01	3.14	1.32	7.45
	Low vs. no exposure to bullying	No vs. high maternal monitoring	0.8	1.16	0.31	4.38	0.88	0.87	0.14	5.55
		Low vs. high maternal monitoring	1	1	0.68	1.47	0	1.91	1.35	2.71
		No vs. high paternal monitoring	0.1	1.91	0.82	4.44	0	4	1.85	8.64
		Low vs. high paternal monitoring	0	1.48	1.01	2.18	0	1.89	1.31	2.73
15	High vs. no exposure to bullying	No vs. high maternal monitoring	0	9.06	2.78	29.47	0.91	1.01	0.84	1.21
		Low vs. high maternal monitoring	0	3.09	1.33	7.18	0.16	1.86	0.78	4.47
		No vs. high paternal monitoring	0.2	2.01	0.64	6.31	0.29	2.27	0.5	10.25
		Low vs. high paternal monitoring	0.4	0.7	0.3	1.63	0.43	1.54	0.53	4.5
	Low vs. no exposure to bullying	No vs. high maternal monitoring	0.5	0.67	0.19	2.37	0	5.19	1.7	15.84
		Low vs. high maternal monitoring	0.1	1.61	0.98	2.63	0	1.92	1.33	2.77
		No vs. high paternal monitoring	0.2	1.86	0.78	4.43	0	2.94	1.52	5.68
		Low vs. high paternal monitoring	0.9	0.98	0.59	1.64	0	2.11	1.34	3.33

## Data Availability

The raw data supporting the conclusions of this article will be made available by the authors upon request.
